# Wordom: A User-Friendly Program for the Analysis of Molecular Structures, Trajectories, and Free Energy Surfaces

**DOI:** 10.1002/jcc.21688

**Published:** 2010-11-29

**Authors:** Michele Seeber, Angelo Felline, Francesco Raimondi, Stefanie Muff, Ran Friedman, Francesco Rao, Amedeo Caflisch, Francesca Fanelli

**Affiliations:** 1 Dipartimento di Chimica, University of Modena and Reggio Emilia v. Campi 18341125 Modena, Italy; 2 Dulbecco Telethon Institute (DTI), University of Modena and Reggio Emilia41125 Modena, Italy; 3 Department of Biochemistry, University of ZurichWinterthurerstrasse 190, CH-8057 Zurich, Switzerland; 4 Freiburg Institute for Advanced Studies (FRIAS), University of FreiburgAlbertstr. 19, 79104 Freiburg, Germany

**Keywords:** structural/dynamics analysis program, free energy landscape, elastic network model, protein structure network, communication paths

## Abstract

Wordom is a versatile, user-friendly, and efficient program for manipulation and analysis of molecular structures and dynamics. The following new analysis modules have been added since the publication of the original Wordom paper in 2007: assignment of secondary structure, calculation of solvent accessible surfaces, elastic network model, motion cross correlations, protein structure network, shortest intra-molecular and inter-molecular communication paths, kinetic grouping analysis, and calculation of mincut-based free energy profiles. In addition, an interface with the Python scripting language has been built and the overall performance and user accessibility enhanced. The source code of Wordom (in the C programming language) as well as documentation for usage and further development are available as an open source package under the GNU General Purpose License from http://wordom.sf.net. © 2010 Wiley Periodicals, Inc. J Comput Chem, 2011

## Introduction

Wordom is a program aimed at fast manipulation and analysis of individual molecular structures and molecular conformation ensembles. Its development started in 2003 and the relative publication appeared in 2007.^1^

A number of programs are already available to analyze molecular structures and dynamics. These include: (a) the most common molecular simulation and analysis packages, like CHARMM,^2^, ^3^ Gromacs,^4^ and Amber^5^, ^6^; (b) a number of molecular viewers, like VMD,^7^ and Pymol^8^; (c) command-line oriented analysis programs and script suites, like MMTSB,^9^ carma,^10^ and pcazip^11^; and (d) packages that provide environments for structural analysis, like Bio3D,^12^ MMTK,^13^ or Biskit.^14^ In this panorama, Wordom was originally conceived as a simple command-line utility to quickly access data in common structural data files. Basic manipulation tools were then implemented, which paved the way for the adoption of a modular framework to easily add analysis routines. At the time of the first publication, novel analysis modules already formed the bulk of Wordom's code, and others have been added since then.

Some of the new modules (Table 1), such as secondary structure assignment (SSA), surface area calculations, and elastic network models (ENM), implement tools that are already available in some form in other software packages or web servers. However, their use on trajectory files is either cumbersome or unpractical. Indeed, programs for SSA and surface computation are widespread, but most of them can only deal with a single structure file at a time, thus making the handling of multiconformation files complex and time consuming. On the same line, ENM can be computed by the CHARMM program^2^, ^3^ or via web servers.^41^–^45^ However, the former is slower and significantly more complicated than Wordom in input setting, whereas the latter do not handle multiconformation files. Moreover, a number of ENM-based analysis tools are available in different programs and/or web servers, whereas Wordom joins many of them together in a single interface.

**Table 1:** New Features in Wordom Since the Original Publication^1^

Module	Label^a^	Function	Reference
Secondary Structure Assignment	SSA	Assignment of secondary structure based on geometric criteria	15, 16
Molecular Surface	SURF	Calculation of solvent accessible, solvent excluding and van der Waals surfaces; surface correlation along a trajectory	17, 18
Elastic Network Model	ENM	Calculation of elastic network models on a protein structure	19–24
Cross Correlation	CORR	Correlations of atomic displacements along a trajectory	25–27
Protein Structure Network	PSN	Calculation of network of amino acid interactions	28–31
PSN Path	PSN-path	Path calculation within protein structure network	32, 33
Clustering	CLUS	Clustering according to conformation similarity	34–36
cut-based Free Energy Profile	cFEP	Computation of a one-dimensional free energy profile that preserves barriers between free energy basins	39, 46
Kinetic Grouping Analysis	KGA	Determination of free energy basins based on kinetic behavior	40

a Abbreviation/acronym used in the text.

Other novel modules introduce procedures and algorithms not available elsewhere, such as protein structure network (PSN) analysis,^28^, ^29^ search for the shortest intra-molecular and inter-molecular communication paths (PSN-path),^32^ kinetic grouping analysis (KGA),^40^ and mincut-based Free Energy Profile (cFEP).^46^ The principles underlying these modules have been reported in the relevant papers, but, so far, no other publicly available software can perform these analyses. In particular, PSN and PSN-path are based on the application of graph theory to protein structures, allowing to represent molecular systems as networks of interacting amino acids and to infer the functional implications of such networks in the context of intra-molecular and inter-molecular communication.^30^, ^31^, ^37^, ^38^, ^47^ Importantly, cFEP and KGA are rigorous methods for determining free energy basins and barriers and thus for investigating the free energy surface of simulated processes, e.g., reversible folding and conformational changes of structured peptides and miniproteins.^39^, ^46^, ^48^

Significant technical improvements include a more user-friendly input syntax and a more general procedure for selecting subsets of atoms. Some parts of the code have been rewritten to gain speed, robustness, and facilitate the addition of new modules. As for performance, Wordom has been modified to treat calculations relative to different frames as different threads and exploit multicore compute architectures (coarse-grained data parallelism). This multithread approach is now present in the modules in which frames are treated independently of each other. Future modules that fall under this category will be able to easily use this kind of threading without major modifications to the code. This approach does not prevent single modules to adopt internal threading. An example is the clustering module, which can now be used in multicore mode in the CPU-intensive step of frame–frame comparison. Finally, an interface with the Python scripting language has been implemented to take advantage of its flexibility and speed of coding.

This article details the analysis tools added to Wordom after the original publication, with particular emphasis on those modules that are not available in other analysis programs.

## New Tools in Wordom

### Secondary Structure Assignment

The SSA module is able to evaluate the secondary structure of a peptide or protein using two methods, DLIKE or DCLIKE, derived from the DSSP^49^ and DSSPcont^15^, ^16^ algorithms, respectively. These two approaches are considered two standards in the field of secondary structure assignments. DSSPcont is a consensus-based DSSP assignment, in which the whole DSSP procedure is run 10 times with different values of the energy cutoff that defines an hydrogen bond (H-bond).^15^, ^16^ Assignments are then weighted according to the cutoff and a consensus is given as the final output. DSSP and DSSPcont assignments are generally comparable.

Both algorithms have been rewritten from scratch since the DSSP license does not allow free reuse of the code. The output is a simple string where the *n*^th^ character corresponds to the secondary structure of the *n*^th^ amino acid. There are eight possible letters in the secondary structure “alphabet”: H, G, I, E, B, T, S, and L, standing for α helix, 3_10_ helix, π helix, extended, isolated β-bridge, hydrogen bonded turn, bend, and unstructured loop, respectively.^15^ No extra information such as that included in the typical DSSP output is given, since the SSA module is meant to be used for a quick analysis of the secondary structure profile along a trajectory, rather than for a complete and throughout characterization of a single structure.

Comparisons between the secondary structure assignments by Wordom and by the DSSP program are shown in Table 2. The agreement is good, i.e., 92%, considering also that most discrepancies do not concern exchanges between helices and strands. The higher speed of the SSA module compared to DSSP shows itself on trajectory files (see Table 3). In fact, whereas the SSA module can compute the secondary structure along a trajectory very fast, DSSP works on single frame files previously extracted from the trajectory. Thus, the better performance of Wordom must be ascribed, at least in part, to the lack of input/output operations associated with handling each molecule conformation as a standalone file (see Table 3). The speedup is more pronounced when dealing with small systems, e.g., peptides.

**Table 2:** Comparison Between the Secondary Structure Assignments Made by Wordom (SSA Module, DLIKE Option) and Those Made by the DSSP Program^a^

	DSSP^a^								
	E	B	T	S	L	H	G	I	Total
Wordom/SSA^a^									
E	2103	31	18	21	85	2	0	0	2260
B	11	58	6	6	38	1	7	0	127
T	16	1	638	51	32	6	3	0	747
S	12	0	5	656	13	0	2	0	688
L	44	3	9	6	1351	3	0	0	1416
H	0	1	11	2	7	951	17	0	989
G	1	0	39	0	3	1	163	0	207
I	0	0	2	0	0	0	0	0	2

Total	2187	94	728	742	1529	964	192	0	6436

a The test set consists of 29 proteins (2CCY, 1ECA, 2IFO, 1TPM, 1HRE, 1PHT, 2POR, 3BCL, 2HLA, 1CDQ, 1AFC, 1MSA, 1VMO, 1HXN, 1NSC, 2BBK, 3AAH, 1TSP, 2PEC, 1PPK, 1STD, 4TIM, 1BRS, 1NTR, 1PYA, 2DNJ, 1PLQ, 1BNH, and 1PYP) selected as representatives of common folds.^50^ Results have been pooled together for each program and compared. Each element *ij* of the matrix reports the number of residues assigned by Wordom and by DSSP to be in conformation *i* and *j*, respectively.

**Table 3:** Speed (in Seconds) Comparison of Secondary Structure Computations

#Residues	#Frames	DSSP_DCD_^a^	DSSP_PDBs_^b^	Wordom_SSA_^c^
316	10,000	1460	920	640
16	10,000	238	155	0.35

a A script extracted each single frame by mean of Wordom and called DSSP on the extracted frame.

b A script called DSSP on the already-extracted frames.

c Calculation through the Wordom SSA module.

Contrarily to DSSP, because Wordom is conceived to operate on the results of simulations, the structure files must contain all the atoms that contribute to the backbone H-bonds. Therefore, structures derived directly from the protein data bank (PDB), especially the X-ray structures that miss hydrogen atoms or entire residues, must be completed before submission to the SSA module.

### Molecular Surface Calculation, Correlation, and Clusterization

Wordom computes different kinds of molecular surfaces using two different algorithms: an exact analytical method developed by Hu and coworkers (i.e., ARVO algorithm)^17^ and a fast numerical method developed by Pascual-Ahuir and coworkers (i.e., GEPOL algorithm).^18^ ARVO calculates the solvent accessible surface area by expressing the molecular surface as surface integrals of the second kind and then transforming these integrals into a sum of double integrals using the stereographic projection method.^17^ In contrast, GEPOL describes the molecular surface as a series of tesserae and then calculates the overall area.^18^ The Wordom implementation of GEPOL allows calculation of the van der Waals, solvent accessible and solvent excluding surfaces as well as tuning of three different parameters [i.e., number of divisions (ndiv), overlapping factor (ofac), and radius of the smaller sphere (rmin)] to balance the speed and accuracy of area computation. Wordom implementations are faster than the original programs (see Table 4).

**Table 4:** Computing Time for Different Modules

Module	# Selected atoms^a^	Approximate CPU time^b^
Surface (Wordom_ARVO_)^c^	115^d^	2980
Surface (ARVO)^e^	115	3690
Surface (Wordom_GEPOL–ASURF_)^f^	115	2130
Surface (GEPOL_ASURF_)^g^	115	2660
Surface (Wordom_GEPOL–ESURF_)^h^	115	5900
Surface (GEPOL_ESURF_)^i^	115	7290
Surface (Wordom_GEPOL–WSURF_)^j^	115	1890
Surface (GEPOL_WSURF_)^k^	115	1970
Correlation (DCC)^i^	360^m^	4
Correlation (LMI)^n^	360	63
PSN^o^	2593	391
PSN-path	–	15 per pair
Clustering (distances only)^p^	316^q^	1461
Clustering (QT-like)^r^	316	100
Clustering (hiero)^s^	316	>50,000
Clustering (leader)^t^	316	10
Clustering (leader)^u^	316	10
Clustering (leader)^v^	316	45

a The considered system is a 10,000 frame trajectory of the GTP-bound Gα_*i*1_ subunit (PDB: 1CIP; 2593 atoms; 316 residues and 1 GTP molecule (44 atoms)).

b CPU time (seconds) on an AMD Athlon 64 3000+, 2 GHz, 2 GB RAM.

c Solvent accessible surface area computed by the Wordom implementation of the ARVO algorithm.

d selection consisted in GTP and first 9 residues (selection /*/@(1–10)/*)

e Solvent accessible surface area computed by the ARVO program.

f Solvent accessible surface area computed by the Wordom implementation of the GEPOL algorithm (highest accuracy).

g Solvent accessible surface area computed by the GEPOL program (highest accuracy).

h Solvent excluded surface area computed by the Wordom implementation of the GEPOL algorithm; accuracy settings: rmin 0.5, ofac 0.8, ndiv 5.

i Solvent excluded surface area computed by the GEPOL program; accuracy setting: rmin 0.5, ofac 0.8, ndiv 5.

j van der Waals surface area computed by the Wordom implementation of the GEPOL algorithm; highest accuracy.

k van der Waals surface area computed by the GEPOL program; highest accuracy.

l Residue-residue correlation by means of the dynamic cross correlation method; masses were not taken into account.

m Selection consisted in all Cα atoms and GTP

n Residue-residue correlation by means of the linear mutual information method; masses were not taken into account.

o PSN analysis probing 11 different *I*_min_ values (from 0.0 to 5.0 with a 0.5 step).

p Only the RMSD-based distance matrix was computed at this stage and written to file.

q All Cα atoms were selected.

r Clustering by the QT-like algorithm, using a precalculated distance matrix (RMSD cutoff 1.0 Å).

s Clustering by the hierarchical algorithm, using a pre-calculated distance matrix (RMSD cutoff 1.0 Å).

t Clustering by the leader-like algorithm (RMSD cutoff 1.0 Å); distance matrix is not necessary.

u Clustering by the leader-like algorithm (RMSD cutoff 1.0 Å) and turning on the non-markovian option. In this case, the bottleneck is disk speed (CPU usage 18%).

v Clustering by the leader-like algorithm (DRMS cutoff 1.0 Å).

Using either one of these two algorithms, Wordom can perform different regression analyses (i.e., linear, logarithmic, exponential, and power) to correlate surface area values from two different selections computed along a trajectory. Moreover, a number of statistical parameters can be derived from the surface timeseries (i.e., range, time average, covariance, and standard deviation). Finally, clustering (binning) of the trajectory snapshots can be also performed on the basis of the surface area values of a given selection, dividing the trajectory frames in different clusters of user-defined width.

### Elastic Network Model

The ENM is a coarse grained normal mode analysis (NMA) technique able to describe the vibrational dynamics of protein systems around an energy minimum. Within this technique, the protein structure is described by a reduced subset of atoms (usually Cα-atoms), whose coordinates can be derived either from structure determinations (crystallography, NMR) or from molecular simulations. The interactions between particle pairs are given by a single term Hookean harmonic potential.^19^ The total energy of the system is thus described by the simple Hamiltonian:


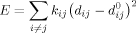
(1)

where *d*_*ij*_ and 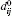 are the instantaneous and equilibrium distances between Cα-atoms *i* and *j*, respectively, whereas *k*_*ij*_ is a force constant, whose definition varies depending on the type of ENM used. The second derivatives of the harmonic potential are stored in a 3N × 3N Hessian matrix (H), whose diagonalization gives a set of 3N-6 nonzero-frequency eigenvectors and associated eigenvalues.

Two alternative versions of ENM have been implemented. In the first version, termed “linear cutoff-enm,” the force constant is equal to 1 for pairwise interactions between the Cα-atoms lying within a cutoff distance chosen by the user, and adjacent Cα-atoms are assigned a force constant equal to 10.^20^ In the second one, termed “Kovacs-ENM,”^21^ the force constant depends on the distance of the interacting particles:


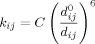
(2)

where *C* is constant (with a default value of 40 Kcal/mol ċ Å^2^).^21^

The structural perturbation method (SPM) has been recently described as a technique useful to characterize allosteric wiring diagrams in the context of the ENM lowest frequency modes.^22^ According to this methodology, amino acid positions that are relevant to protein dynamics are searched by perturbing systematically all the springs that connect the Cα-atoms and then measuring the residue-specific response of such perturbations in the context of a given mode *m*. The perturbation response is computed as:


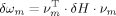
(3)

where ν_*m*_ is the eigenvector of mode *m*, 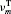 is its transpose, and δ*H* is the Hessian matrix of the perturbation to the energy of the elastic network:


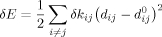
(4)

**Figure 1:**
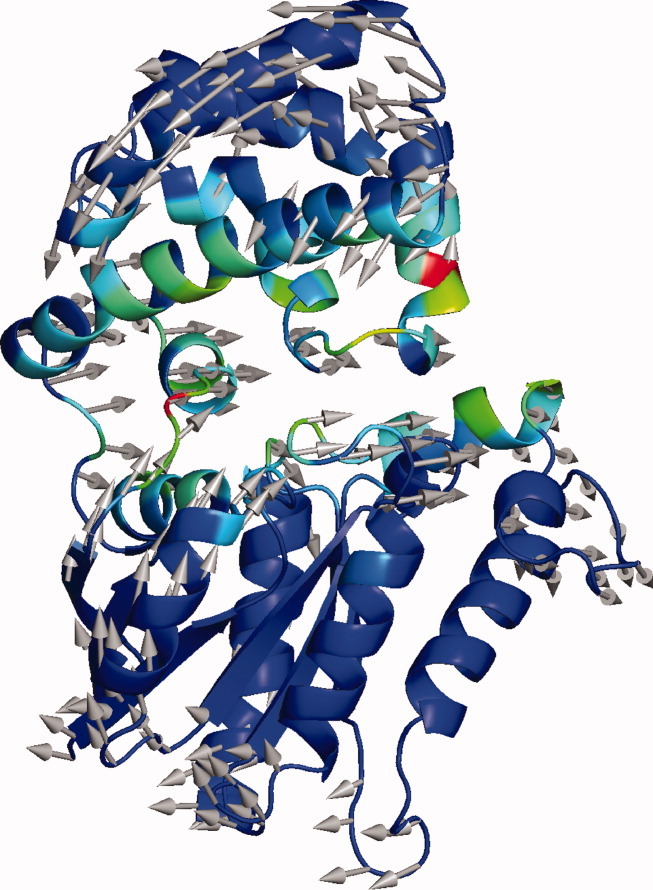
Application of the SPM (within the ENM module) to the GTP-bound Gα_*i*1_-subunit (PDB: 1CIP). Each Cα-atom is colored according to the response to the perturbation of the 1^st^ normal mode. Coloring from red to blue indicates maximum (100%) and minimum (0%) perturbations, respectively. Arrows point in the direction of the 1^st^ normal mode. [Color figure can be viewed in the online issue, which is available at wileyonlinelibrary.com.]

The response δω_*im*_ is proportional to the elastic energy of the springs that are connected to the *i*^th^ residue when they are perturbed by an arbitrary value (0.1), thus defining the most critical nodes for the dynamics of a given mode. The number of modes used for the computation is specified by the user (from 1 up to 3N-6). It is also possible to generate, for each analyzed mode, a pdb file containing the values of δω_*im*_ in the β-factor field (Fig. 1).

Theoretical β-factors can be computed inside the ENM module, by the formula^23^


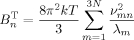
(5)

where ν_*mn*_ is the *n*^th^ element of eigenvector *m*, λ_*m*_ is the associated eigenvalue, *k* is the Boltzmann constant, and *T* is the temperature in K.

Cross correlations between theoretical and experimental β-factors can be also computed according to the following equation:


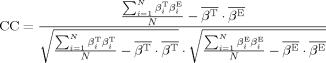
(6)

where 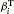 and 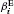 are the theoretical and experimental β-factors, and 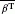 and 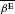 are the theoretical and experimental β-factor average over all atoms, respectively. The number of modes used for the computation is specified by the user (from 1 up to 3N-6).

Moreover, involvement coefficients *I* between the ENM modes and the displacement vector between a given structure/frame T and a reference structure R can be computed according to the following equation:


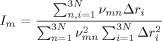
(7)

where 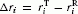 and 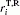 is the *i*^th^ coordinate in the two conformers and ν_*mn*_ is the *n*^th^ element of eigenvector *m*.^24^ By default, the computation is done for all 3N-6 modes, and only the values of *I* greater than an arbitrary threshold (i.e., 0.2) are output.

The cumulative square overlap (CSO) between all modes and the displacement vector is computed according to the following equation:


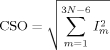
(8)

Finally, residue correlation *C*_*ij*_ is computed as:^51^


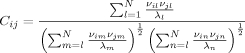
(9)

### Cross Correlation

Wordom implements two different algorithms to calculate correlations of atomic displacements along an MD trajectory. One algorithm, called dynamic cross-correlation (DCC),^25^ is a simple and well established method based on the calculation of the normalized covariance of atom/residue position vectors. DCC values are computed as:


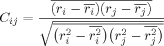
(10)

where *i* and *j* may be atoms or centroids of atoms grouped by residue, and *r*_*i*_ and *r*_*j*_ are the corresponding position vectors. DCC represents the extent of atom/residue displacement correlation within a range that goes from 1.0 to −1.0; where 1.0 indicates completely correlated (same period and phase) and −1.0 completely anti-correlated (same period and opposite phase) displacements. The second algorithm, called linear mutual information (LMI),^26^, ^27^ is computationally more expensive (see Table 4) than DCC but overcomes some limitations of the DCC algorithm and is able to estimate correlations between perpendicular motions. LMI values are computed as:


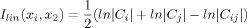
(11)

where *i* and *j* may be atoms or residues, *C*_*ij*_ is the pair-covariance matrix, and *C*_*i*_ and *C*_*j*_ are marginal covariance matrices.^26^, ^27^ LMI correlation values can vary from 0.0 to 1.0, which indicate completely uncorrelated and completely correlated displacements, respectively.

The Wordom implementation of the DCC and LMI algorithms incorporates some setup options. In particular, it is possible to calculate correlations by treating atoms independently or collectively with respect to the residues they belong to. It is also possible to take into account the atomic masses.

**Figure 2:**
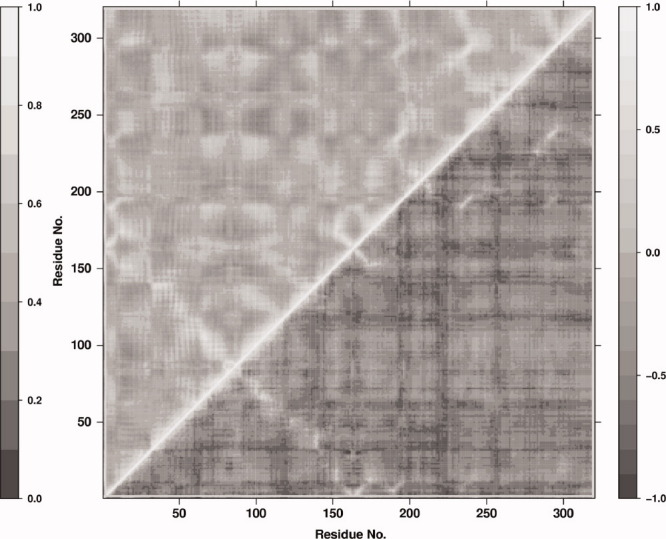
Cross-correlation matrix of the atomic fluctuations of the Gα_*i*1_-subunit Cα-atoms and the geometrical center of GTP. The regions below and above the matrix main diagonal concern the DCC and LMI correlation methods, respectively. DCC correlation values go from −1.0 (fully anti-correlated motions) to 1.0 (fully correlated motions), whereas LMI correlation values go from 0.0 (fully uncorrelated motions) to 1.0 (full correlated motions).

For a selection of 360 atoms within 10000 trajectory frames, the DCC and LMI methods took, respectively, 4^″^ and 63^″^ on the same processor (Table 4). The relative correlation matrices are shown in Figure 2.

### Protein Structure Network

In recent times, the concept of PSN has been explored, giving more insights into the global properties of protein structures.^30^, ^31^ The representation of protein structures as networks of interactions between amino acids has proven to be useful in a number of studies, such as protein folding,^47^ residue contribution to the protein–protein binding free energy in given complexes,^37^ and prediction of functionally important residues in enzyme families.^38^ All these aspects pertain to the issue of intra-molecular and inter-molecular communication.^30^, ^31^

Wordom implementation of PSN analysis is based on the work and algorithms described in the relevant papers by the Vishveshwara and coworkers.^28^, ^29^ PSN is constructed from the atomic coordinates of residues, which represent the nodes of the network. Two nodes are connected by an edge if the percentage of interaction between them is greater than or equal to a given Interaction Strength cutoff.


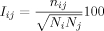
(12)

where *I*_*ij*_ is the interaction percentage of nodes *i* and *j*, *n*_*ij*_ is the number of side-chain atom pairs within a given distance cutoff (4.5 Å as a default), and *N*_*i*_ and *N*_*j*_ are, respectively, the normalization factors (NF) for residues *i* and *j*, which take into account the differences in size of the different nodes and their propensity to make the maximum number of contacts with other nodes in protein structures. The NFs for the 20 amino acids in our implementation were taken from the work by Kannan and Vishveshwara.^28^ Novel NFs for nonamino acid nodes can be introduced as well by the user. In this respect, the current version of the module holds also the NFs for retinal, guanine nucleotide di- and tri-phosphates (GDP and GTP, respectively), and Mg^2+^. In detail, retinal NF (i.e., 170.13) was computed as the average number of contacts done by the molecule in a dataset of 83 crystallographic structures concerning the different photointermediate states of bacteriorhodopsin, bovine rhodopsin, sensory rhodopsin, and squid rhodopsin. The NFs for GDP and GTP (i.e., 220.19 and 274.78, respectively) were derived from datasets of 55 and 69 G proteins, respectively. Finally, the NFs for Mg^2+^ concerns GTPases and is 14.65 and 22.01 in the GDP- (i.e., based upon 41 GTPase structures) and GTP-bound states (i.e., based upon 68 GTPase structures). *I*_*ij*_ are calculated for all node pairs excluding *j* = *i* ± *n*, where *n* is a given neighbour cutoff (2 as default), and each node pair with an *I*_*ij*_ value greater than or equal to a given *I*_min_ cutoff is connected by an edge. Different networks can be achieved by probing a range of *I*_min_ cutoffs. At high *I*_min_ cutoffs, only nodes with high number of interacting atom pairs will be connected by edges, indicative of stronger inter-residue interactions. At a given *I*_min_ cutoff, those nodes that realize more than a given number of edges (4 as default) are called hubs. The percentage of interaction of a hub node is


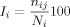
(13)

where *I*_*i*_ is the hub interaction percentage of node *i*, *n*_*ij*_ is the number of side-chain atom pairs within a given distance cutoff and *N*_*i*_ is the normalization factor of residue *i*. Node inter-connectivity is finally used to highlight cluster-forming nodes, where a cluster is a set of connected amino acids in a graph. Node clusterization procedure is such that nodes are iteratively assigned to a cluster if they can establish a link with at least one node in such cluster. A node not linkable to existing clusters initiates a novel cluster and so on until the node list is exhausted. The size (defined as the number of nodes) of the largest cluster is used to calculate the *I*_critic_ value. *I*_critic_ is defined as the *I*_min_ at which the size of the largest cluster is half the size of the largest cluster at *I*_min_ = 0.0. At *I*_min_ = *I*_critic_ weak node interactions are discarded, emphasizing the effects of stronger interactions on PSN properties.

The Wordom implementation of PSN analysis allows the user to: (a) modify all the involved cutoffs (i.e., distance, neighbor, hub); (b) make residue selections; (c) set *I*_min_ ranges; and (d) set, when dealing with a trajectory, the fraction of frames for which a PSN property is defined as stable. Furthermore, Wordom computes all network properties described in the relevant papers by Vishveshwara's group (i.e., interaction strength for all node pairs, stable node interactions, hub frequencies, cluster compositions, and dimensions).^28^, ^29^

**Figure 3:**
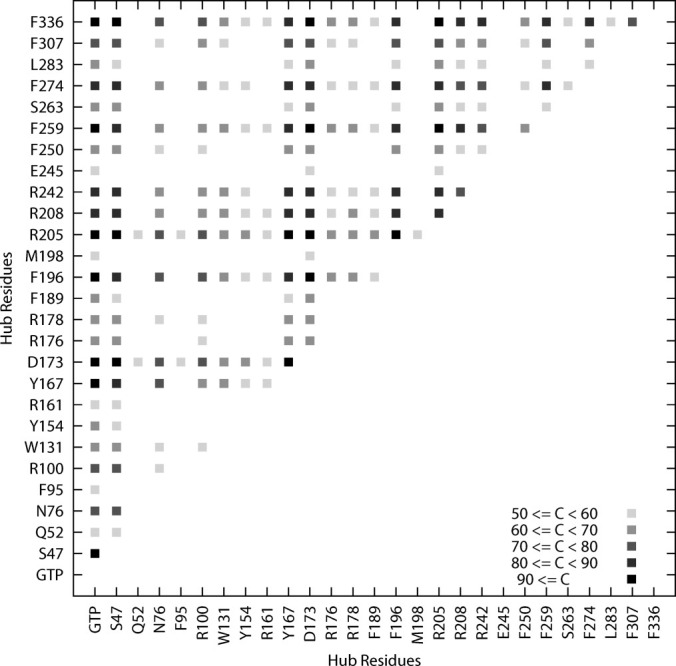
Hub correlation analysis on a 10 ns MD trajectory of GTP-bound Gα_*i*1_-subunit. Each dot corresponds to two amino acids that show a correlated tendency to behave as hubs (i.e., that are syncronized in their hub behavior in more than 50% of the trajectory frames). An *I*_min_ = 3.0% was employed for the PSN analysis.

An original feature of Wordom is the hub correlation analysis, a simple but effective method to highlight correlations in the propensity of two nodes to behave as hubs along an MD trajectory (Fig. 3).

**Figure 4:**
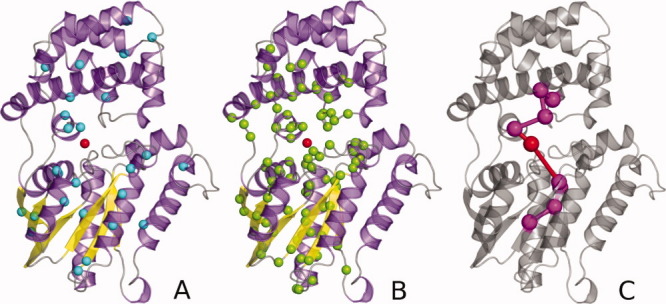
Results of PSN and PATH analyses on a 10 ns MD trajectory of GTP-bound Gα_*i*1_-subunit. (A) Cα-atoms of the 27 stable hub residues, at *I*_min_ = 3.0%, are represented as cyan spheres. The GTP molecule, which is a stable hub as well, is shown as a red sphere centered on the C4^′^ ribose atom. Nodes are considered as stable hubs if they are involved in at least four connectivities at a given *I*_min_ (3.0% in this case) in more than 50% of the trajectory frames. (B) The 90 nodes that contribute to the largest cluster at *I*_min_ = 3.0% are shown as green spheres centered on the Cα-atoms. The GTP molecule, which participates as well in such cluster, is shown as a red sphere centered on the C4^′^ ribose atom. (C) Representation of the most frequent shortest communication path (i.e., frequency = 46%). The amino acids that participate in the path are shown as magenta spheres centered on the Cα-atoms, whereas GTP, which participates in the path as well, is shown as a red sphere centered on the C4′ ribose atom. The two apical residues in this path are A152 and I222, located, respectively, in the α-helical and Ras-like domains.

The results of an application of the PSN module to a 10,000 frame trajectory of the Gα_*i*1_-subunit complexed with GTP are shown in Figures 4A and 4B. The relative CPU time required for such a demonstrative calculation is reported in Table 4.

#### Search for Communication Paths

As an extension of the PSN analysis tool, Wordom can calculate the shortest non-covalently connected path(s) between two residues of interest in a single structure or in a trajectory (Fig. 4C), by combining PSN node inter-connectivities and residue correlated motions, as described in the relevant paper by the Gosh and Vishveshwara.^32^ Path search through the PSN-path module uses Dijkstra's algorithm^33^ to traverse PSN inter-connectivities, and to find the shortest paths in each frame. It consists in: (a) search for all shortest paths between selected residue pairs based upon the PSN connectivities and (b) selection of paths that contain at least one residue correlated with either one of the two extremities (i.e., the first and last amino acids in the path). Once the shortest paths have been found, their frequencies, i.e., the number of frames containing the selected path divided by the total number of frames in the trajectory, are computed, which helps selection of the most meaningful paths. Steps (a) and (b) of path search employ the outputs from the PSN and CORR modules, respectively. The Wordom implementation allows the user to tune several parameters of the path-search routine (i.e., minimum interaction strength cutoff between nodes, lowest accepted residue correlation cutoff, minimum length and frequency of paths). Either the DCC or LMI methods can be chosen as a source of residue correlations.

### Clustering

The original RMSD- and DRMS-based clustering module allowed the choice of three different algorithms: leader-like,^34^ hierarchical^35^ and quality threshold-like (QT-like).^36^ QT-like differs from the original QT algorithm in the check performed to assess whether a conformation belongs to a cluster or not. The original QT builds a perspective cluster for each frame by comparing it with all others and adding conformations progressively farther away from the starting frame until each new addition is within the chosen threshold with respect to all previously added frames. The largest of all these perspective clusters is then taken as the first cluster, its members are taken out of the conformation population and the procedure is run again until all conformations are either in a cluster or isolated. The threshold can be seen as the diameter of the cluster thus formed. In contrast, QT-like builds the clusters only checking that newly added conformations are within the threshold with respect to the reference frame; the threshold is thus the radius of the cluster.

The clustering module has been optimized both in its performance (speed and memory usage) and accuracy. In the leader-like^34^ algorithm (the fastest but least accurate one), each subsequent frame is compared with the existing cluster centers and, in case no cluster center is within the threshold, a new cluster is created with the frame as its center. The original implementation allowed the choice of two different frame-comparison modalities. According to the first modality a frame is compared with all the existing clusters and assigned to the nearest one (more accurate, default behavior). With the second option a frame is assigned to the first cluster within the threshold (faster). In the latest version a third option has been added, such that each frame *n* is compared with the existing clusters moving backward from the cluster that holds frame *n* − 1, to the cluster that holds frame *n* − 2, and so on, until a distance lower than the threshold is found. In non-Markovian data sets (e.g., snapshots of MD simulations saved every few ps which are correlated) this approximation greatly speeds up the process, because the likelihood that a frame belongs to the same cluster as the preceding frame(s) is quite high. The accuracy of the new option is only slightly lower than the “comparison with all clusters” approach, but the execution is faster than the original “stop at first viable cluster” option. Leader-like clustering is less accurate than the QT or the Hierarchical algorithms since it compares each frame only with the clusters that have been already found along the trajectory, thus making the outcome dependent on the frame order.

More relevant improvements concern the Hierarchical and QT-like algorithms. Indeed, they have been both modified so that the original memory requirements have been almost halved. Furthermore, the actual clustering algorithms massively use multithreading in the CPU-intensive computation of the inter-frame distances (RMSD or DRMS). Also, the distance matrix can now be saved for later use, so that, if clustering with different threshold values is desired, the distance-computing step needs to be performed only once. Finally, the original two-pass clustering has been improved as well. In detail, after a first clustering run on a subset of frames, a second pass can be run that assigns each considered frame to the nearest cluster found in the first run. In the original version, frames with no clusters within a distance lower than the threshold were labelled as isolated. In contrast, Wordom now treats these isolated frames as new cluster centers, so that new clusters can be found and populated in the second run. This improves the overall accuracy and allows for a smaller portion of the total data set to be used in the first run.

When accuracy is paramount, the QT-like algorithm is probably the most appropriate, being more accurate than the leader-like one and significantly faster (with comparable accuracy) than the Hierarchical algorithm (Table 4). Yet, in spite of the improvements, it remains considerably memory-hungry. Therefore, when dealing with big data sets (>1M–10M frames, depending on the available computing power and memory), with which it is impossible to consider all frame–frame distances, the user can choose to either use QT on a subset of frames and then run a two-pass clustering, or to opt for the leader-like algorithm.

### Determination of Free Energy Basins and Barriers

Wordom has two distinct modules, cFEP^46^ and KGA,^40^ devoted to the identification of (meta)stable states sampled by MD simulations. The key idea of cFEP and KGA is to group conformations not according to structural criteria, but mainly according to equilibrium kinetics. In this way, an analysis of the MD trajectory in terms of free energy basins, i.e., basins of attraction of the dynamics, is provided. The main advantage of cFEP with respect to KGA is the information on the height and location of the free-energy barriers along the cumulative partition function,^46^ which can be used to identify the transition state structure(s).^52^ On the other hand, the KGA procedure does not require the use of a reaction coordinate to determine the free-energy basins.^40^

For both cFEP and KGA procedures, MD snapshots (i.e., Cartesian coordinate sets) need to be finely clusterized and assigned to a discrete set of microstates. Clusterization can be done according to atomistic, i.e., RMSD-based clustering, or to coarse-grained representations such as secondary structure strings. Both RMSD-based and coarse-grained clustering have proven to be good discretization methods of MD trajectory snapshots into a set of microstates that describe large conformational changes (see Ref.^40^, ^48^, and^53^–^56^ for examples in protein folding). Application to large proteins requires more sophisticated clustering procedures like principal component space.^57^

#### Mincut-Based Free Energy Profile

The cFEP module refers to a rigorous method introduced by Krivov and Karplus^46^ for determining a one-dimensional free energy profile that preserves the barriers between free energy basins; given the barriers, free energy basins can be determined. The method uses the relative partition function,^46^ which is a reaction coordinate that takes into account all possible pathways to a reference state (e.g., the folded state).

**Figure 5:**
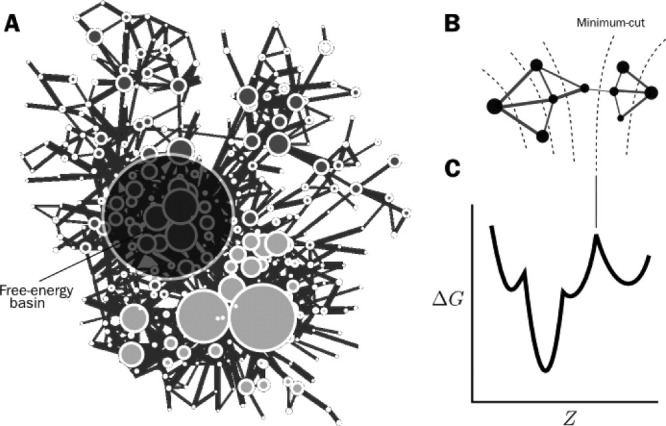
Complex network analysis of free energy landscapes. (A) Conformation space network. Nodes and links are protein conformations (i.e., microstates, see main text) and direct transitions sampled during the MD simulation, respectively. Node size is proportional to the population of the microstate, whereas link width is proportional to the transitions frequencies, i.e., larger link widths indicate more frequent transitions. Densely connected regions of the network represent rapidly interconverting microstates that belong to the same free energy basin (highlighted by a shaded circle). (B) Simplified example of a two state system. The free energy barrier between the two macro-states is represented by a region of minimum flow in the network (identified by a minimum-cut). (C) Cut-based free energy profile (cFEP). The free energy is projected onto the partition function-based reaction coordinate *Z*, a projection that preserves the barriers as it takes into account all possible pathways to a reference microstate.^46^ The solid vertical line indicates the correspondence between the minimum-cut and the highest free energy barrier.

The cFEP algorithm is based on a network description of the conformational dynamics. Each microstate (see above) represents a node of the conformation space network^53^, ^58^ and a link is made if a direct transition between two microstates is observed during the timeseries in a time step of a given size (see Fig. 5).^59^

**Figure 6:**
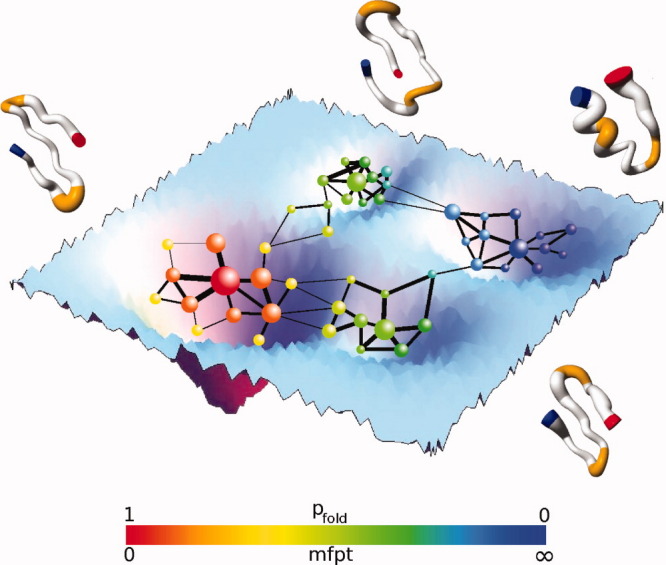
Network description of MD and evaluation of kinetic distance. The high-dimensional free-energy surface is coarse-grained into nodes of a network. The figure shows a schematic illustration of the transition network of a β-sheet peptide where nodes represent microstates and links represent direct transitions sampled along the MD simulation(s). The size of the nodes and links is proportional to the statistical weight of the microstates and number of transitions, respectively. The cFEP method implemented in Wordom requires a reference microstate. In this simplified illustration, the reference microstate is the large red sphere in the center of the folded state (which is the β-sheet structure, i.e., the basin on the left). The kinetic distance of each node from the reference microstate can be evaluated in Wordom by the folding probability (*p*_fold_) or the mean first passage time (mfpt). The kinetic distance is rendered by the continuous coloring from red (folded, i.e., *p*_fold_ = 1 or mfpt = 0) to blue (unfolded, i.e., *p*_fold_ = 0 or mfpt = infinity).

The cFEP module implemented in Wordom is a precise and fast approximation of the minimum-cut method.^60^, ^61^ The free energy is projected as a function of the partition function relative to a reference node.^39^, ^46^ With this method, microstates are ranked according to their kinetic proximity with respect to a reference microstate (Fig. 6). The relative partition function is used as the progress coordinate, and the free energy barriers are determined as a function of it, either based on the probability of reaching the folded state before unfolding (*p*_fold_)^46^ or on the mean first passage time (mfpt)^39^ to a selected node (both calculated analytically from the transition matrix). The *p*_fold_ implementation, which requires a target node, is appropriate to find barriers between two well-defined basins, which are specified by the user through the assignment of *p*_fold_ = 1 to the representative node of one basin, and *p*_fold_ = 0 to the representative node of the other. On the other hand, the mfpt-based method is more suitable for free energy profiles relative to a single target basin (e.g., for unfolding profiles), for which the representative node of the target basin is assigned mfpt_target_ = 0.

#### Kinetic Grouping Analysis

The free energy basins are determined by KGA on the basis of kinetic behaviors (fast relaxation at equilibrium) along an MD simulation.^40^ The KGA method is based on a network description of the conformational dynamics.

Two protein microstates are grouped in a basin if, along the MD trajectory, they interconvert frequently within a short commitment time τ_commit_, which represents a typical relaxation time within basins. The principle behind this approach is that if two conformations interconvert rapidly, they are not separated by a barrier and therefore belong to the same basin. The τ_commit_ is a characteristic of the investigated system. It is an important (user-chosen) parameter of KGA and defines the resolution with which basins are isolated. A short τ_commit_ will group structures only locally or if the free energy surface is very smooth. A longer τ_commit_ is more generous and might group sub-basins, isolated by a shorter τ_commit_, into larger basins. The log–log plot of the distribution of first passage times to the native microstate (or a representative microstate of another basin) usually reflects two timescales: the inter- and intra-basin relaxation times (see Fig. 7 of Muff and Caflisch^40^). The barrier that separates the two regimes can give a good indication for the relaxation time.

The KGA module allows for isolation of either all relevant basins at once or of a single basin. In the first case, for a fixed commitment time τ_commit_, a matrix with interconversion (commitment) probabilities *p*_commit_ between any pair of microstates can be calculated in principle, and microstates with *p*_commit_ ≥ 0.5 are grouped together. Because the computational cost of all-against-all calculations increases quadratically, in practice one selects a subset of highly populated microstates (e.g., the 500 most populated microstates), calculates the *p*_commit_-matrix and divides them into basins. In a post-processing step, all other microstates are assigned commitment probabilities to the isolated basins; finally, all microstates having a *p*_commit_ ≥ 0.5 to a given basin are assigned to it. Otherwise, the microstates remain unassigned. Both the heavy-microstate calculation and the post-processing are done by Wordom in the same function. On the other hand, if only one basin is of interest or if the relaxation times within basins lay on different timescales, it is better to choose an appropriate τ_commit_ for each basin separately and then calculate the commitment probability (*p*_commit_) according to it. In this way, it is possible to isolate basins one-by-one. In this case, the user has to run the procedure a number of times equal to the number of basins that need to be isolated. In addition to the τ_commit_, a representative microstate of each basin (usually the most populated microstate in the basin) has to be specified. Finally, all microstates that commit to the representative microstate of a basin with probability *p*_commit_ ≥ 0.5 are considered as part of that basin.

## Python Bindings

Using the SWIG (simple wrappers and interface generator)^62^ tools, a python module has been written that gives access to most of Wordom's input/output functions and structures in the python environment via a simple import command. Basic analysis functions (e.g., RMSD, distances, atoms selections) are also exposed to the python environment. The availability of Wordom's input/output functions allows scripts to operate directly on molecular data, whereas access to Wordom's analysis functions makes it easy to compute properties on molecules or whole trajectories, and to further process the output without writing full-fledged C code or resort to temporary files. It is also practical to write the prototype of an analysis module in python and then convert it to C to enhance its performance, as has been done for some of the recently added modules.

## Conclusions

Wordom is a user-friendly program for manipulating and analysing data from structural studies and molecular simulations. The latest release represents a significant improvement and enrichment of the original version published in 2007,^1^ as it provides new analysis tools that are unique to Wordom. These include new procedures for efficient structural analysis such as dynamic PSN and shortest communication path modules, which are effective tools to infer amino acids essential for stability and function as well as to unravel intra-molecular and inter-molecular communication mechanisms. Other novelties are user-friendly methods for determining free energy basins and barriers using the network of transitions sampled by MD simulations. With these new tools, Wordom can be used to analyze the free energy surface and therefore investigate the thermodynamics and kinetics of complex molecular processes, e.g., the reversible folding of structured peptides (Fig. 6).

Improvements include also the implementation of an interface with the popular scripting language Python.

Like the original version, this version of Wordom is released under the general purpose license (GPL), which allows anybody to download, modify, and redistribute both source code and binary files. This license has been adopted in order to foster diffusion and encourage contributions to the code.
